# From antibiotic to chalkophore: the biology and evolution of SF2768 in *Streptomyces*

**DOI:** 10.1042/EBC20250022

**Published:** 2026-08-19

**Authors:** Kenji Ueda

**Affiliations:** Life Science Research Center, College of Bioresource Sciences, Nihon University, 1866 Kameino, Fujisawa 252-0880 Japan

**Keywords:** chalkophore, copper, evolution, secondary metabolism, SF2768, Streptomyces

## Abstract

Copper availability is tightly regulated in microbial environments, yet the diversity and evolutionary origins of copper-chelating metabolites remain poorly understood. SF2768, a diisonitrile compound from *Streptomyces*, functions both as a broad-spectrum antibiotic and as a highly specific secreted copper chelator (chalkophore) that binds copper with a 1:2 stoichiometry. Its biosynthesis depends on an NRPS encoded in the *sfa* gene cluster, which also includes an ABC transporter required for uptake of the Cu–SF2768 complex. A phylogenetic survey revealed that while some *Streptomyces* species retain both biosynthetic and uptake genes, others possess only the uptake system, indicating interspecies utilization of the metabolite. These findings suggest that SF2768 may have originated as an antibiotic that kills competing microbes by inducing copper starvation, and was later co-opted by certain *Streptomyces* as a copper acquisition system. The distribution of *sfa* genes illustrates how novel metabolic functions can emerge from secondary metabolism and become ecologically embedded. SF2768 provides a model for understanding the evolutionary transition of secondary metabolites from competitive weapons to cooperative or utilitarian factors within microbial communities.

## Introduction

Antibiotics are compounds that inhibit the growth of bacteria or other microorganisms [1]. Extensive screening efforts over the past decades have revealed their remarkable structural and functional diversity. The availability of diverse antibiotics has formed the foundation not only of modern medicine but also of fundamental research in molecular biology. Advances in biosynthetic studies have provided deep insights into how complex molecular architectures are assembled within cells. In parallel, chemical biology approaches have expanded our understanding of cellular processes by using bioactive compounds as chemical probes.

This review focuses on the roles of antibiotics in natural environments. Since the seminal question “Are antibiotics naturally antibiotics?” posed by the late Professor Julian Davies [2], accumulating evidence has shown that many antibiotics exert biological activities distinct from growth inhibition [3]. A deeper understanding of the functional diversity of microbial natural products is therefore expected to provide important ecological and evolutionary insights into the organization of microbial ecosystems. Here, interactions mediated by copper availability are discussed, based on the dual nature of a *Streptomyces* metabolite that functions both as an antibiotic and as a copper acquisition factor.

**Figure 1:**
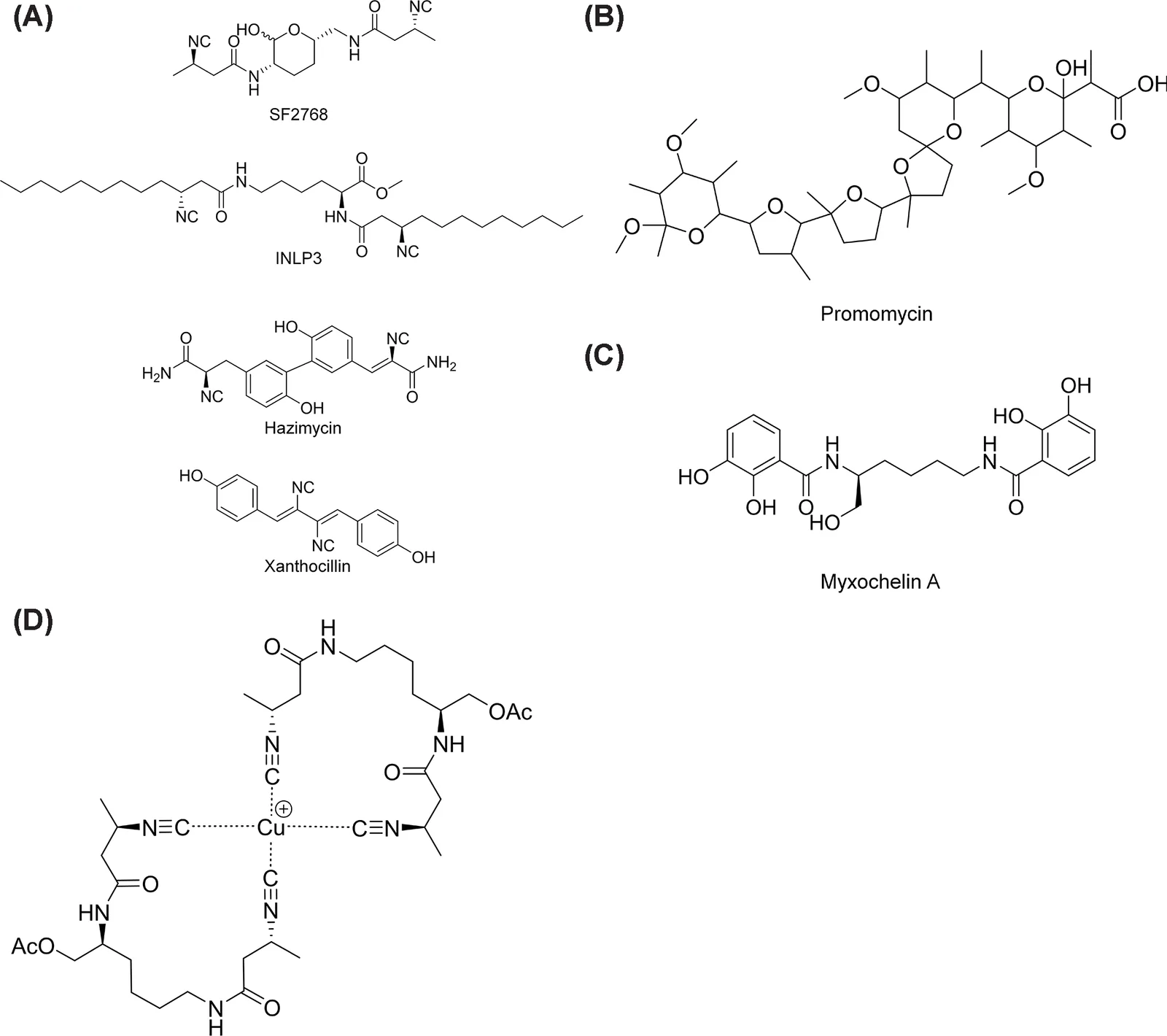
Chemical structure of the compounds described in this paper (**A**) Diisonitrile chalkophores. SF2768 (*S. griseorubiginosus* and *S. thioluteus*), INLP3 (*M. fortuitum*), Hazimycin (*K. purpeofusca*), and Xanthocillin (*P. notatum* and *A. fumigatus*) are shown. (**B**) Promomycin, the inducer of SF2768, produced by *S. scabrisporus.* (**C**) Myxochelin A, a siderophore produced by *S. aurantiaca*. (**D**) The copper-bound form of an SF2768 derivative (diisonitrile acetate) determined by the NMR titration analysis [12].

The diisonitrile compound SF2768 (Figure 1A) was originally discovered by Sho-ichi Amano and colleagues at Meiji Seika Pharmaceutical Co. in the 1990s [4]. The compound was isolated from the culture broth of a *Streptomyces* species based on its fungicidal activity. It inhibited fungi including the pathogenic yeasts *Candida albicans* and *Cryptococcus neoformans*, as well as both Gram-positive and Gram-negative bacteria.

Later, S. Amano rediscovered the same compound in the author’s laboratory at Nihon University [5]. We were studying the effects of cocultivation between different *Streptomyces* species on their antibiotic production [6]. We found that growing a colony of *Streptomyces scabrisporus* strain 153 near *Streptomyces griseorubiginosus* strain 574 induced the latter to produce a bactericidal substance [7].

The stimulatory molecule produced by *S. scabrisporus* was purified and identified as a polyether compound named Promomycin (Figure 1B) [7]. Although Promomycin itself exhibited antibiotic activity, its stimulatory effect occurred even at subinhibitory concentrations, indicating that it acts as a signaling molecule inducing the otherwise cryptic antibiotic production in *S. griseorubiginosus*.

S. Amano isolated the inducible antibiotic whose production was triggered by Promomycin and demonstrated that it was identical to SF2768 [5]. Although the precise mechanism remains unknown, these findings suggest that SF2768 is an inducible metabolite produced in response to environmental cues. Because Promomycin is a polyether, the signal may reflect membrane-structure perturbation.

**Figure 2:**
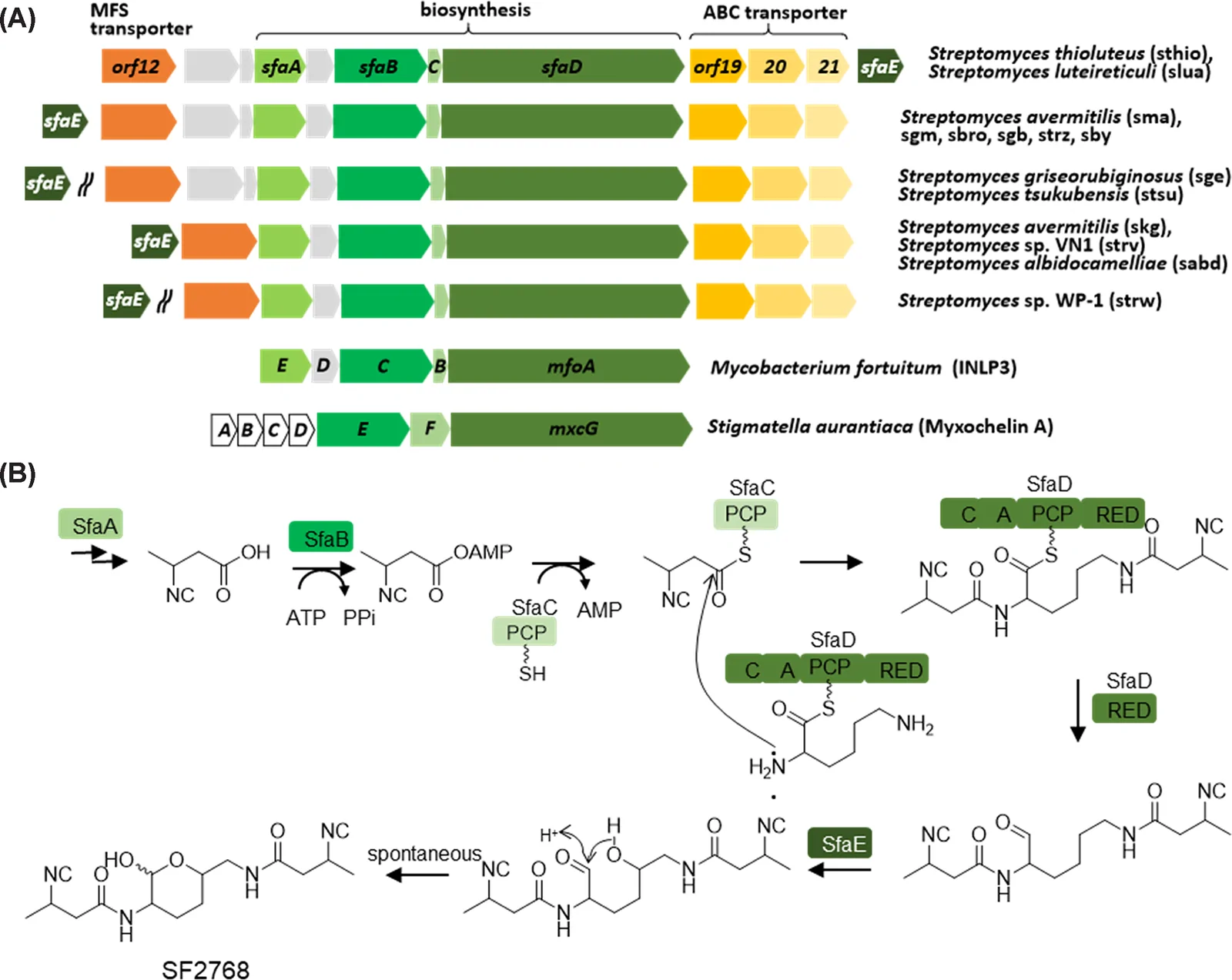
Genes and pathway of SF2768 biosynthesis (**A**) Schematic representation of the *sfa* genes involved in the synthesis of SF2768 of *S. thioluteus* and the related gene clusters distributed to 13 *Streptomyces* spp. The 5 strains retaining the second type cluster shown by their organism codes are: sgm, *Streptomyces aurantiacus*; sbro, *Streptomyces broussonetiae*; sgb, *Streptomyces globisporus*; strz, *Streptomyces* sp. 71268; and sby, *Streptomyces buecherae*. The *mfoA* cluster for INLP3 synthesis in *M. fortuitum* and the *mxc* cluster for Myxochelin A production in *S. aurantiaca* are also shown. (**B**) Biosynthetic pathway of SF2768 proposed by Wang et al. [8].

In 2017 [8], Wang et al. published a landmark study elucidating the biosynthesis and function of SF2768. They identified the *sfa* gene cluster (Figure 2A) responsible for SF2768 biosynthesis in *Streptomyces thioluteus* and demonstrated that SF2768 is synthesized through reactions catalyzed by enzymes encoded therein, including an NRPS. The resulting SF2768 functions as a secreted copper chelator that mediates copper uptake from the environment into the cell.

## Mechanisms relating to SF2768

### Biosynthesis

The *sfa* biosynthetic gene cluster of *S. thioluteus* (Figure 2A) consists of the *sfaABCD* biosynthetic operon and flanking transporter genes (*orf12* and *orf19-20-21*) [8]. Based on amino acid sequence similarity between SfaBCD and MxcEFG of *Stigmatella aurantiaca*—well-studied enzymes involved in the biosynthesis of the siderophore Myxochelin A (Figure 1C) [9–11]—Wang et al. characterized the biosynthetic pathway of SF2768 and proposed the scheme shown in Figure 2B [8].

SfaA (a putative dioxygenase) generates 3-isocyanobutanoic acid. The building block is adenylated by SfaB (an AMP ligase), and the acyl chain is transferred to SfaC (a peptidyl carrier protein). SfaD (NRPS), which prefers lysine as a substrate, mediates condensation of lysine with the SfaC-bound acyl intermediate followed by reductive release. The intermediate is hydroxylated by SfaE, and subsequent spontaneous cyclization generates SF2768.

### Transport

The *sfa* cluster encodes two types of membrane transporters. *orf12*, located upstream of *sfaA*, encodes a major facilitator superfamily (MFS) transporter. Because MFS transporters frequently mediate antibiotic efflux, Orf12 is thought to export SF2768; deletion of *orf12* reduces extracellular SF2768 [8].

The three genes downstream of the cluster (*orf19-20-21*) encode subunits of an ABC transporter. Orf19, the substrate-binding protein, shows strong similarity to the iron(III)-siderophore binding proteins of ferric-siderophore uptake systems. Deletion of this ABC transporter decreases intracellular Cu(I)-SF2768 [8], indicating that it imports copper-bound SF2768. By analogy with siderophores for iron uptake, secreted small molecules that selectively bind and transport copper are referred to as chalkophores.

### Copper binding

Biochemical analyses by Wang et al. [8] demonstrated that purified SF2768 forms a complex with either Cu(I) or Cu(II), but not with other metal ions such as Fe(II) or Fe(III), indicating high copper specificity. The detailed structural analysis by Xu and Tan [12] using an acetate derivative of SF2768 revealed that copper binding occurs with a 1:2 (Cu:SF2768) stoichiometry (Figure 1D).

At the time of these discoveries, knowledge of chalkophores was extremely limited [13]. Methanobactin, produced ribosomally with post-translational modifications in methanotrophic bacteria such as *Methylosinus trichosporium*, was one of the few well-studied examples [14–16]. Copper uptake involving Coproporphyrin III in *Paracoccus denitrificans* [17,18] and Cu(II) binding by Yersiniabactin [19–21] were also known.

Recently, the number of identified copper-chelating compounds has increased [22]. These include dityrosine-based diisonitriles such as Hazimycin (produced by *Streptomyces* spp. and *Kitasatospora purpeofusca*) [23] and Xanthocillin (from *Penicillium notatum* and *Aspergillus fumigatus*) (Figure 1), all initially discovered as antibiotics.

Another notable group of diisonitrile compounds is the isonitrile lipopeptides (INLPs) produced by pathogenic mycobacteria [24,25]. These compounds have been implicated in metal transport associated with bacterial virulence. Identification and characterization of their biosynthetic gene clusters revealed striking similarities to the *sfa* cluster [26]. Detailed studies by Buglino et al. [26], using synthetic diisonitrile chalkophores, demonstrated that these compounds also function in copper acquisition under copper-limiting conditions.

During preparation of this manuscript, Jia et al. [27] identified a new INLP chalkophore INLP3 (Figure 1A) from *Mycobacterium fortuitum* and characterized its copper-binding properties in detail. The *mfo* gene cluster (Figure 2A) responsible for INLP3 biosynthesis shows marked similarity to the *sfa* cluster and other INLP biosynthetic clusters in mycobacteria [26]. Notably, however, mycobacterial clusters lack genes encoding an ABC transporter system.

### Antibiotic activity

SF2768 was originally identified through its ability to inhibit fungal growth as well as both Gram-positive and Gram-negative bacteria [4]. This inhibitory effect can be rescued by adding excess copper, demonstrating that its antibiotic activity results from copper sequestration [8].

Recently, Futamura et al. [28] showed that SF2768-treated *C. albicans* cells fail to undergo budding or hyphal branching, defects that are restored by copper supplementation. Thus, SF2768 likely perturbs copper-dependent developmental pathways in fungi, suggesting potential utility as both a research tool and a therapeutic candidate.

Copper chelation by SF2768 reduces bioavailable copper, impairing diverse copper-dependent primary metabolic pathways such as respiration. This explains its broad antimicrobial spectrum.

Zhu et al. [29] reported that copper sequestration by SF2768 can lead to ROS accumulation. Because copper deprivation inactivates copper-dependent phenol oxidases such as tyrosinase, the formation of melanin and other antioxidants is suppressed, increasing ROS levels. This mechanism may further contribute to the broad antibiotic activity of SF2768.

## Phylogenetic assessment of gene distribution

### Biosynthetic genes

A search of the KEGG complete genome database (https://www.kegg.jp/) identified *sfa*-like biosynthetic gene clusters resembling that of *S. thioluteus* (Figure 2A) in the genomes of 13 *Streptomyces* species. The synteny of these clusters was strictly conserved, except that *sfaE* was located at a different genomic position in 10 *Streptomyces* species, including *Streptomyces avermitilis* (Figure 2A).

**Figure 3:**
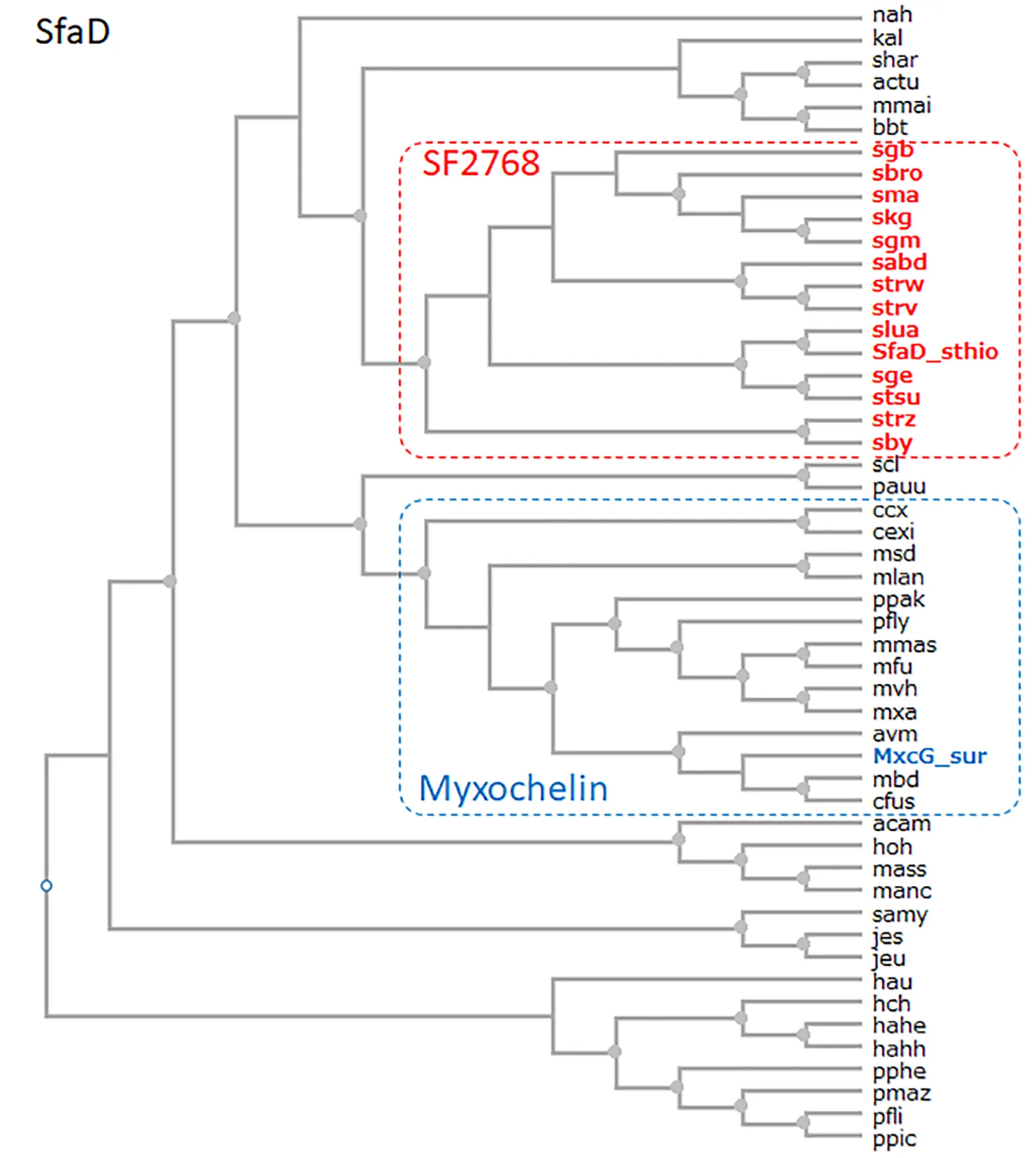
Phylogenetic relationship of SfaD homologs The tree (maximum-likelyhood tree) was drawn by using the tree option of KEGG (https://www.kegg.jp/) under default settings. SfaD_Sthio and MxcG_sur indicate SfaD of *S. thioluteus* and MxcG of *S. aurantiaca*, respectively. Other 3- or 4-letter shown at each branch end is the organism code of the bacterial organism retaining the homologous protein registered in the genome sequence database. The 14 *Streptomyces* spp. retaining the whole Sfa gene cluster are shown in red and bold letters. Nodes supported by a high (>80) SH(Shimodaira Hasegawa test)-like local support values are indicated by closed circles.

Figure 3 shows the phylogenetic relationships among the top 50 SfaD homologs identified in the KEGG database. The tree clearly separates into two distinct clades: one containing SfaD from *S. thioluteus*, and the other containing MxcG from *S. aurantiaca*. The former clade consists exclusively of SfaD orthologs from the 13 aforementioned *Streptomyces* species. This suggests that NRPS enzymes belonging to this clade have specifically evolved to synthesize SF2768 or structurally related molecules. A similar clade pattern was observed among the top 50 SfaA homologs (data not shown).

In contrast, the NRPS clade containing MxcG consisted exclusively of Gram-negative bacteria belonging to the family *Myxococcaceae*. These organisms also formed a distinct clade in the phylogenetic tree of MxcD homologs (data not shown). Thus, NRPS enzymes in this clade likely direct the biosynthesis of Myxochelin or structurally related ferric-chelating compounds.

### Uptake genes

**Figure 4:**
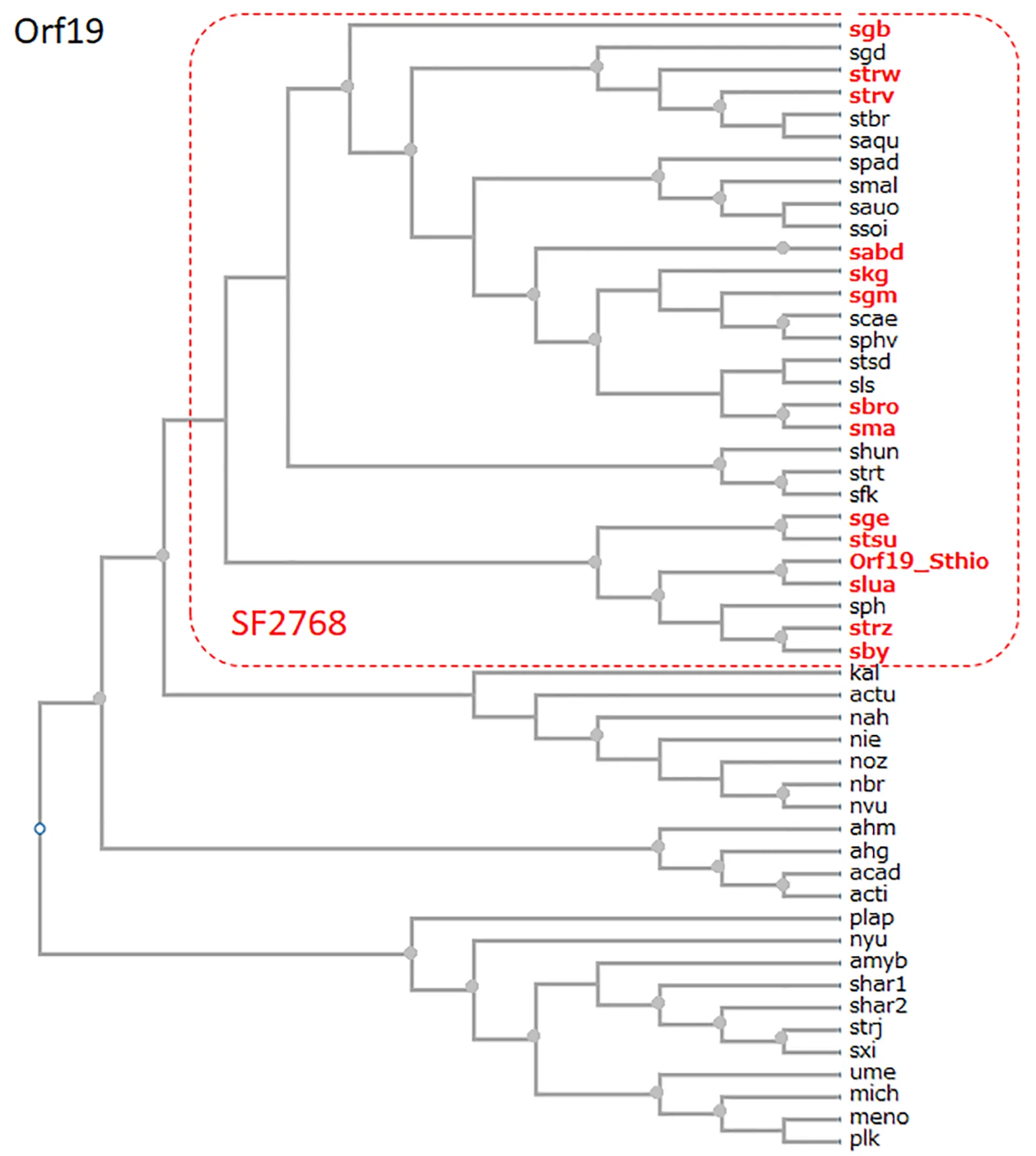
Phylogenetic relationship of Orf19 homologs Conditions of tree construction are the same as that appear in Figure 3 legend. Orf19_Shtio corresponds to the Orf19 protein of *S. thioluteus*. shar1 and shar2 indicate the two homologs of *Streptomyces harbinensis* NA02264 whose coding sequence ID are shar_HUT13_23865 and shar_HUT13_09625, respectively.

Figure 4 presents the phylogenetic relationships among the top 50 Orf19 homologs, the predicted substrate-binding domain of the ABC transporter responsible for uptake of the copper-bound SF2768 dimer. Orf19 is expected to recognize Cu–SF2768 at the cell surface and deliver it to the membrane transporter complex formed by Orf20 and Orf21.

The major clade formed by *Streptomyces* homologs includes not only the Orf19 orthologs from the 13 *sfa*-positive species but also homologs from *Streptomyces* strains lacking the *sfa* biosynthetic genes. Presumably, these organisms do not synthesize SF2768 but are still capable of importing SF2768 produced by other *Streptomyces*.

A similar but much broader example of such “public” metallophore utilization is known for desferrioxamines [30–33], siderophores synthesized by *Streptomyces* and a few other bacteria but utilized by a wide range of microbes—including fungi—that cannot synthesize them. This indicates that some siderophores act as global symbiotic factors.

In contrast to the widespread distribution of ferrioxamine-utilizing proteins, Orf19 homologs are limited to *Streptomyces*, suggesting that the ecological range of SF2768 utilization is narrow. This may reflect the relatively high environmental abundance of soluble copper compared with iron. Even so, *Streptomyces* may require elevated copper acquisition to sustain their complex developmental cycle. As reported for ferrioxamine uptake systems [30,34], Orf19 may exhibit broad substrate specificity, potentially recognizing not only SF2768 but also related diisonitrile–copper complexes.

### Significance of copper in *Streptomyces* development

We previously reported the widespread presence of a conserved copper-utilization operon, *sco* (*Streptomyces* copper utilization operon), in *Streptomyces* genomes [35]. Although its detailed function remains unclear, Sco proteins are predicted to form a membrane-bound copper acquisition complex. ScoC, an Sco1/SenC family copper chaperone containing an N-terminal secretion signal, likely binds extracellular copper and transfers it to other components of the complex.

Several studies have demonstrated the significant impact of copper availability on *Streptomyces* development. T. Kieser [36] first reported that copper supplementation enhances sporulation efficiency in *Streptomyces lividans*. We subsequently observed that developmental and antibiotic production defects of *Streptomyces tanashiensis* were rescued by copper supplementation [37]. Similar phenotypes were observed in *scoC* mutants of several model *Streptomyces* strains [35]. These observations suggest that the copper-dependent activity of cytochrome oxidase is fundamental to *Streptomyces* development [38].

The Sco1/SenC family was originally identified as a factor required for cytochrome oxidase assembly in yeast (“Sco” meaning “synthesis of cytochrome oxidase”). Today, this family is known to be broadly conserved among microorganisms and its biochemical roles have been characterized in several systems. While the primary function of *Streptomyces* ScoC may also involve copper delivery to cytochrome oxidase, its extracellular localization and association with multiple membrane proteins suggest a broader importance for copper acquisition in these complex developmental bacteria.

### Insights into the role of secondary metabolism

Nouioui et al. [39] reported that SF2768 production is stimulated by SARP (*Streptomyces* antibiotic regulatory protein), a transcriptional activator that specifically controls antibiotic biosynthetic genes. This observation supports the classification of SF2768 as a secondary metabolite whose production is developmentally regulated and largely decoupled from primary metabolism. At the same time, its function as a chalkophore implies a direct contribution to copper-dependent primary metabolic processes.

The author speculates that secondary metabolism acts as an evolutionary testing ground. Because it is largely decoupled from primary metabolism, organisms can safely explore diverse chemical innovations without risking essential biological functions. Among such experimental metabolites, some may later acquire useful physiological roles, aided by the evolution of specialized utilization mechanisms, ultimately becoming specialized metabolites linked to primary metabolism. If such functions are broadly beneficial, they may become disseminated across microbial populations.

The distribution of *sfa* genes may illustrate such a maturation process. The copper-acquisition function of SF2768 may have originated within the realm of secondary metabolism and later become widespread because of its utility and/or to mitigate the otherwise toxic effects of the compound.

An alternative, but not mutually exclusive, hypothesis is that SF2768 initially evolved as an antibiotic that killed competing microorganisms by depleting environmental copper. This model accounts for the SARP-dependent regulation of *sfa* expression. The organization of INLP biosynthetic clusters in mycobacteria, which lack transport genes, may represent an ancestral state. Later, some *Streptomyces* species evolved the ability to import Cu–SF2768, allowing them to exploit it as a copper source. Ultimately, 14 *Streptomyces* species integrated both biosynthetic and uptake mechanisms into a complete *sfa* cluster.

Recently, Matsuda et al. [23] identified the biosynthetic gene cluster for Hazimycin (Figure 1) in *Kitasatospora purpeofusca*. Like the *sfa* cluster, the Hazimycin cluster contains both biosynthetic and transporter genes, suggesting that similar evolutionary processes may apply to other chalkophores in actinomycetes.

Currently, utilization of SF2768 appears restricted to certain *Streptomyces* species, possibly reflecting the high copper demand associated with their complex life cycle. However, future environmental changes may increase the benefit of using extracellular copper packages. In such circumstances, SF2768 could emerge as a broader symbiotic factor. SF2768 thus exemplifies a microbial innovation originally developed for competitive interactions but later repurposed for cooperative or communal functions.

## Summary

- Recent years have seen increasing recognition of the structural diversity of chalkophores, copper-binding metallophores.
- SF2768, a diisonitrile metabolite of *Streptomyces* originally characterized for its broad antibiotic activity, is synthesized by NRPS-dependent pathways and binds copper extracellularly.
- Copper sequestration by SF2768 is toxic to many microbes, yet beneficial to those that have evolved the ability to import and utilize the Cu–SF2768 complex.
- The distribution of SF2768-related genes provides insight into how new functions—initially arising within secondary metabolism—can evolve into versatile mechanisms with ecological significance.

## Open Access

Open access for this article is provided via our Subscribe to Open programme, made possible through the support of our subscribing institutions. Learn more here: https://portlandpress.com/pages/open_access_options_and_prices#conditional.

## Abbreviations

cds: protein coding sequence
INLP: isonitrile lipopeptide
MFS: major facilitator superfamily
NRPS: non-ribosomal peptide synthase
SARP: *Streptomyces* antibiotic regulatory protein
ROS: reactive oxygen species
